# From comparative oncology to AI-enabled precision medicine: translational biomodels for triple-negative breast cancer

**DOI:** 10.3389/fonc.2026.1896728

**Published:** 2026-08-12

**Authors:** Tiago Veiras Collares, Fabiana Kömmling Seixas, Claudia Pessoa, Maria Lucia Zaidan Dagli

**Affiliations:** 1 Molecular and Cellular Oncology Research Group, Cancer Biotechnology Laboratory, Technological Development Center, Federal University of Pelotas, Pelotas, Brazil; 2 Department of Pathology, School of Veterinary Medicine and Animal Science, University of São Paulo - USP, São Paulo, Brazil; 3 INCT T-Bio2: Translational Biodiscovery and Biomodels, CNPq, Fortaleza, Brazil; 4 Department of Physiology and Pharmacology, Faculty of Medicine, Federal University of Ceará, Fortaleza, Brazil

**Keywords:** artificial intelligence, canine mammary tumors, comparative oncology, genetically engineered mouse models, Oncopig, organoids, patient-derived xenografts, precision medicine

## Abstract

Triple-negative breast cancer (TNBC), defined by the absence of estrogen receptor, progesterone receptor, and human epidermal growth factor receptor 2 expression or amplification, comprises a biologically heterogeneous group of tumors with aggressive clinical behavior and limited biomarker-guided treatment options. Although chemotherapy, immune-checkpoint inhibition, poly(ADP-ribose) polymerase inhibitors, and antibody–drug conjugates have expanded the therapeutic landscape, durable benefit remains constrained by genomic instability, homologous recombination deficiency, phenotypic plasticity, immune–stromal interactions, and treatment-driven evolution. This Perspective critically examines how complementary translational biomodels can be organized into a fit-for-purpose framework for TNBC precision oncology. Spontaneous canine mammary tumors provide naturally evolving disease in immunocompetent hosts, whereas patient- and species-derived organoids enable scalable functional perturbation and drug-response profiling. Patient-derived xenografts preserve clinically relevant tumor heterogeneity and treatment-selected states, while genetically engineered mouse models support mechanistic interrogation of defined oncogenic events *in vivo*. Large-animal platforms, including the Oncopig Cancer Model, may add anatomical, procedural, and longitudinal realism; however, their application to TNBC remains emergent and requires disease-specific validation. We further propose a patient–model–algorithm–patient reverse-translational loop in which histopathology, radiology, molecular profiles, and functional response data are integrated through computational pathology, radiomics, multi-omics factor models, and supervised multimodal learning. Robust implementation will require patient-level data partitioning, external validation, calibration, explainability, and explicit control of batch effects, domain shift, and interspecies bias. Rather than prioritizing a single experimental system, TNBC precision medicine should rely on coordinated evidence across models with distinct and complementary strengths and limitations. This integrated strategy may improve biomarker prioritization, therapeutic hypothesis testing, and selection of clinically actionable interventions.

## TNBC biology, therapeutic evolution, and the translational gap

Triple-negative breast cancer (TNBC) is defined by the absence of clinically meaningful estrogen receptor and progesterone receptor expression and by the lack of human epidermal growth factor receptor 2 overexpression or amplification. This operational definition is useful for treatment selection, but it does not describe a single biological entity. Transcriptomic and genomic analyses identified basal-like, mesenchymal, luminal androgen receptor, immunologically active, and other states with distinct pathway dependencies and drug sensitivities (1–3). More recent single-cell and spatial studies further show that TNBC heterogeneity is distributed across malignant-cell metaprograms, immune and stromal cell states, and their spatial organization. A 2026 analysis of 427,857 cells from 101 treatment-naive tumors identified four patient-level archetypes, 13 malignant-cell metaprograms, 49 immune and stromal states, and eight multicellular ecotypes associated with chemotherapy response (4). Imaging mass cytometry has likewise shown that response to immune-checkpoint blockade depends not only on the abundance of individual cell types, but also on proliferating CD8-positive T cells, major histocompatibility complex class II-positive cancer cells, and specific cancer-immune spatial interactions (5).

These observations have practical consequences for biomodel design. A model that reproduces tumor-cell genotype but lacks the relevant immune architecture may be adequate for target dependency testing and inadequate for immunotherapy prediction. Conversely, a naturally occurring tumor with intact host immunity may provide ecological validity but insufficient experimental control to establish causality. TNBC also evolves under treatment through clonal selection, phenotypic plasticity, epithelial-mesenchymal transitions, altered DNA-damage repair, and remodeling of the tumor microenvironment. Early recurrence is more frequent than in hormone receptor-positive disease, while pathological complete response after neoadjuvant therapy is strongly associated with improved long-term outcome (6, 7). Residual disease is therefore not simply a smaller version of the pretreatment tumor; it is a treatment-selected biological state that should be modeled explicitly.

The therapeutic landscape has become similarly stratified. In stage II-III TNBC, neoadjuvant chemotherapy combined with pembrolizumab followed by adjuvant pembrolizumab improved overall survival in KEYNOTE-522 (8). In advanced disease, pembrolizumab plus chemotherapy improved overall survival in tumors with a programmed death ligand 1 combined positive score of at least 10 (9). Adjuvant olaparib improves invasive disease-free survival in high-risk, human epidermal growth factor receptor 2-negative breast cancer carrying germline BRCA1 or BRCA2 pathogenic variants (10), while capecitabine can reduce recurrence in patients with residual disease after neoadjuvant chemotherapy (11). Sacituzumab govitecan established the clinical value of an antibody-drug conjugate targeting trophoblast cell-surface antigen 2 in metastatic TNBC (12). Thus, model systems must now address biomarker-defined questions involving homologous recombination deficiency, immune context, residual disease, target expression, drug delivery, and acquired resistance rather than merely reproducing rapid tumor growth.

The central translational problem is consequently one of evidence integration. No single platform simultaneously provides naturally evolved disease, controlled perturbation, intact immunity, patient-linked heterogeneity, longitudinal access, and human-scale procedures. A robust TNBC program should therefore assign each model a defined question, prespecified readout, and validation role.

## Comparative and patient-derived models: biological fidelity versus experimental control

Spontaneous canine mammary tumors provide a distinctive comparative entry point because they arise in outbred, immunocompetent animals exposed to shared environmental, hormonal, metabolic, and age-related influences. Genomic studies have identified conserved copy-number alterations and driver pathways between canine simple mammary carcinomas and human breast cancer, while also revealing canine-specific biology, particularly in complex and mixed tumors (13). Whole-exome and transcriptome analysis of 191 canine mammary tumors identified frequent PIK3CA alterations, broad phosphoinositide 3-kinase-AKT pathway dysregulation, and a basal-like subgroup with epithelial-mesenchymal transition and poor prognosis, but also a relative scarcity of ERBB2 amplification (14). Cross-species transcriptomic analyses further support a canine basal-like subtype that clusters with human basal-like breast cancer (15). These data justify comparative biomarker discovery, but they also argue against treating all canine triple-negative tumors as direct equivalents of human TNBC.

The principal limitations of the canine setting are clinically important. Histological complexity, reproductive history, breed structure, variable diagnostic work-up, nonuniform treatment, and differences in receptor assessment can introduce substantial confounding. The term ‘triple negative’ should therefore be accompanied by harmonized immunohistochemical thresholds, histological classification, genomic or transcriptomic subtype assignment, treatment annotation, and outcome definitions. Prospective veterinary clinical studies should incorporate pretreatment and on-treatment sampling whenever feasible, but therapeutic decisions must remain centered on animal welfare and veterinary benefit.

Organoids convert human or canine tumors into experimentally tractable systems while preserving a meaningful fraction of tumor architecture and molecular heterogeneity. A human breast cancer organoid biobank demonstrated retention of histology, receptor status, copy-number profiles, sequence alterations, and subtype diversity, and supported drug screening linked to xenograft and clinical responses (16). Canine mammary tumor organoids similarly maintained morphology, receptor status, genomic alterations, and drug-response patterns and were compatible with CRISPR-Cas9 perturbation (17). Matched patient-derived xenograft and organoid platforms have extended this strategy by coupling scalable ex vivo screening with *in vivo* confirmation and, in selected cases, real-time functional precision oncology (18).

Organoids nonetheless impose selection. Establishment efficiency varies among tumors; media composition and extracellular matrices can favor particular epithelial states; clonal proportions may drift with passage; and standard cultures incompletely reproduce vasculature, pharmacokinetics, endocrine context, and immune-stromal interactions. Their best use is therefore not as miniature patients, but as functional assays for cell-intrinsic dependencies, combination prioritization, resistance evolution, and genetically controlled perturbation. Co-cultures, air-liquid interface systems, autologous immune components, and matrix standardization can increase biological breadth, but each addition also increases analytical variability. Concordance should be tested against the tumor of origin at early passage and, whenever possible, against patient or animal response.

### *In vivo* models: preserving evolution and testing causality

Patient-derived xenografts (PDXs) preserve patient-linked tumor architecture, clonal heterogeneity, metastatic behavior, and therapy-selected states more effectively than conventional cell-line xenografts. Breast cancer grafts can reproduce pathology, growth, metastasis, and clinically relevant outcomes (19), while large PDX collections have enabled compound screening across heterogeneous tumors (20). PDXs are especially valuable for testing residual disease, metastatic progression, and treatment sequences when matched pretreatment and post-treatment material is available. However, engraftment is selective, latency may be incompatible with individual clinical decisions, human stroma is progressively replaced by murine stroma, and standard hosts are immunodeficient. These constraints limit direct inference about immune-checkpoint therapy and can distort stromal and pharmacological relationships. Humanized hosts partly address immune questions but add donor mismatch, graft-versus-host effects, cost, and variability.

Genetically engineered mouse models (GEMMs) offer a complementary form of resolution. By controlling lineage, timing, and oncogenic events in an immunocompetent host, GEMMs can establish causal relationships between drivers, cell of origin, immune context, progression, and therapeutic response. Cross-species transcriptomic analyses have identified conserved features between murine mammary tumors and human breast cancer (21) and mapped individual GEMMs to human molecular subtypes (22). Yet GEMMs can overrepresent the initiating engineered lesion, develop on restricted genetic backgrounds, and progress through evolutionary paths that differ from sporadic human TNBC. A model should therefore be selected according to the biological mechanism under study rather than by the broad label ‘breast cancer model’.

The strongest design couples these platforms. Organoids provide rapid perturbation and dose-response testing; PDXs evaluate whether patient-linked architecture and evolution alter the result; and GEMMs determine whether a defined driver or immune mechanism is causal. Discordance across models should be treated as data rather than failure, because it can reveal whether a proposed biomarker depends on immune context, stromal species, exposure kinetics, or a selected clone.

### Large-animal models and the unresolved human-scale validation gap

Large-animal platforms address questions that cannot be reproduced adequately in rodents, including clinical-scale imaging, serial image-guided biopsy, catheter-based delivery, surgical workflow, device testing, repeated pharmacokinetic sampling, and radiological-pathological correlation. The Oncopig Cancer Model carries Cre-inducible KRASG12D and TP53R167H transgenes and was established as a spatially and temporally controllable porcine cancer platform (23). Porcine mammary systems have also been explored through BRCA1-related engineering and transformation of porcine mammary epithelial cells (24).

A critical distinction is required, however. The KRAS/TP53 Oncopig is not currently a validated breast cancer or TNBC model. KRAS mutation is uncommon in human breast cancer, and published large-animal reviews explicitly identify the absence of a fully established porcine breast cancer model (25). The Oncopig should therefore be positioned as an emerging human-scale validation layer rather than as a disease-equivalent TNBC platform. Its near-term roles include testing imaging signatures, biopsy schedules, locoregional delivery, procedural feasibility, and longitudinal sampling strategies after a candidate has been prioritized in disease-specific systems. Its longer-term relevance to TNBC will depend on mammary-restricted induction, breast cancer-relevant genetic drivers, orthotopic xenografts, or other implementations that reproduce mammary tumor biology.

The value of this scale is demonstrated by organ-specific Oncopig studies in which tumors supported human-sized catheters and image-guided interventions, as shown in pancreatic cancer (26). Limitations remain substantial: high cost, specialized facilities, fewer animals, complex breeding and induction, longer experimental timelines, and comparatively limited availability of porcine-specific reagents. Large-animal studies should consequently be reserved for questions in which anatomy, procedure, serial access, or whole-organism scale is itself part of the hypothesis.

### From “AI” to an explicit multimodal analytical framework

**Figure 1:**
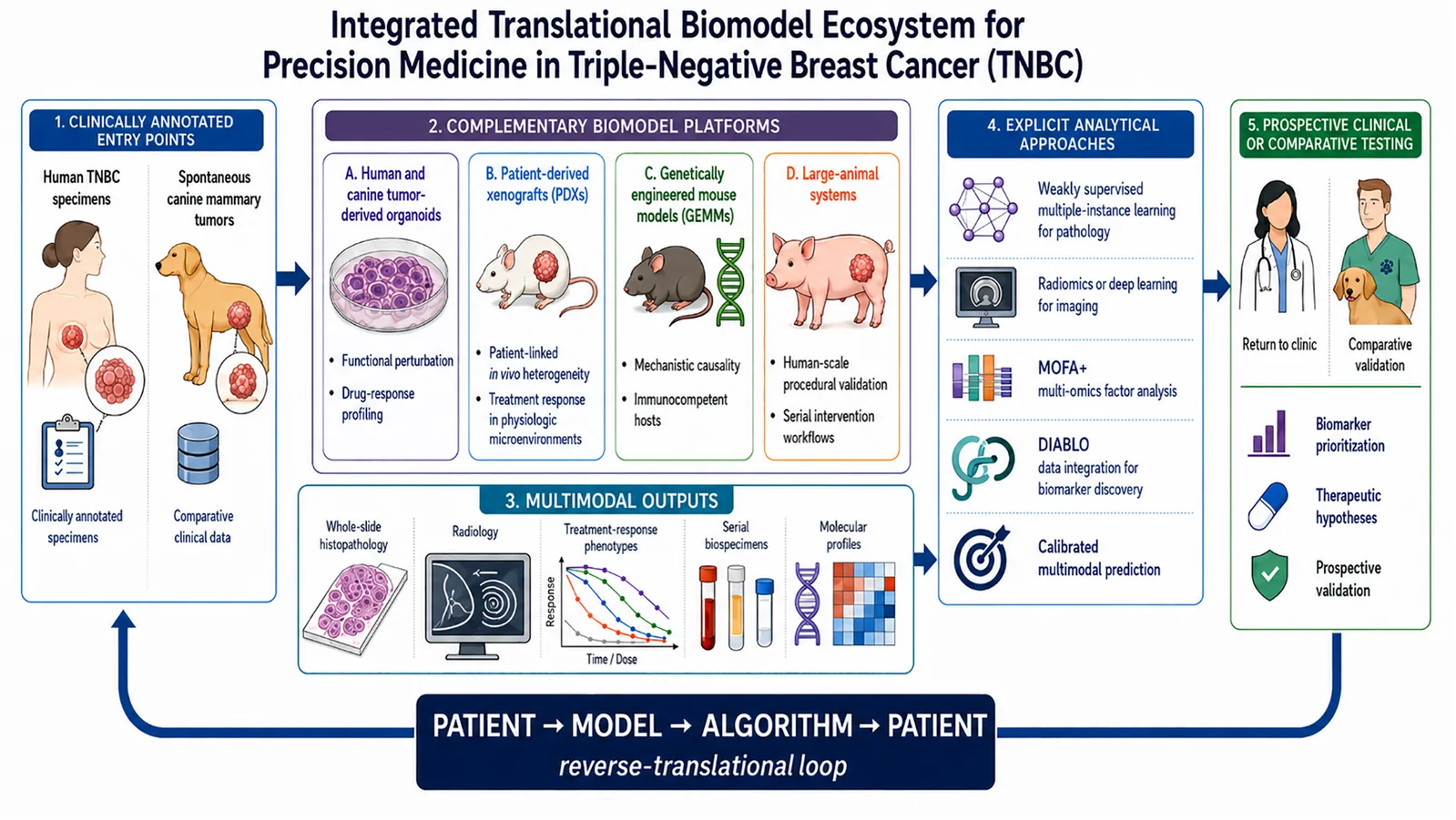
Integrated comparative and translational biomodel ecosystem for precision medicine in TNBC. Human TNBC specimens and spontaneous canine mammary tumors provide clinically annotated entry points. Complementary platforms contribute different forms of evidence: human and canine tumor-derived organoids support perturbation and drug-response profiling; patient-derived xenografts preserve patient-linked *in vivo* heterogeneity; genetically engineered mouse models test mechanistic causality in immunocompetent hosts; and large-animal systems provide human-scale procedural validation. Multimodal outputs include whole-slide histopathology, radiology, treatment-response phenotypes, serial biospecimens, and molecular profiles. Explicit analytical approaches may include weakly supervised multiple-instance learning for pathology, radiomics or deep learning for imaging, MOFA+ (Multi-Omics Factor Analysis) and DIABLO (Data Integration Analysis for Biomarker discovery using Latent cOmponents) for unsupervised multi-omics factors, and calibrated multimodal prediction. The resulting hypotheses return to prospective clinical or comparative testing through a patient-model-algorithm-patient loop.

Artificial intelligence (AI) should describe a specified analytical method, not a generic integration box. In a TNBC biomodel ecosystem, the computational workflow begins with a prespecified clinical or translational endpoint: pathological complete response, residual cancer burden, progression, metastatic competence, target dependency, or concordant drug response across models. Data are then organized by patient or animal, specimen, time point, model lineage, treatment, and assay. This hierarchy is essential because random splitting of image tiles, organoids, or serial samples can leak information from the same biological source into training and test sets and produce misleading performance (**Figure 1**).

For digital pathology, hematoxylin and eosin-stained whole-slide images can be analyzed with convolutional neural networks or vision transformers. Because slide-level labels are more available than exhaustive region annotations, weakly supervised multiple-instance learning is particularly relevant. Clustering-constrained attention multiple-instance learning (CLAM), for example, identifies informative regions while generating attention maps that can be reviewed by pathologists (27). Breast cancer studies have shown that deep-learning models can infer programmed death ligand 1 status from routine histology with external validation (28) and predict spatial gene expression from histological images (29). In the proposed cross-species setting, models should first be trained and validated within species, followed by domain-adaptation or transfer-learning experiments and leave-one-species-out tests. Stain normalization, scanner metadata, tissue quality, and species-specific morphology must be modeled explicitly rather than treated as nuisance variation.

Radiological data require a parallel but distinct pipeline. Conventional radiomics extracts standardized shape, intensity, and texture features from segmented lesions, whereas deep learning can learn features directly from magnetic resonance imaging, computed tomography, ultrasound, or positron emission tomography. A recent TNBC study combined intratumoral and peritumoral vascular radiomic features to predict pathological complete response (30). For biomodel translation, image acquisition should use harmonized sequences and phantoms where possible, with segmentation reproducibility, feature stability, and scanner effects quantified before model fitting. Longitudinal delta-radiomics can then be linked to serial pathology and molecular measurements.

Multi-omics integration should likewise match the study objective. Multi-Omics Factor Analysis version 2 (MOFA+) provides an unsupervised latent-factor model for discovering shared and modality-specific sources of variation (31). Data Integration Analysis for Biomarker discovery using Latent cOmponents (DIABLO), implemented in mixOmics, performs supervised integration and selects correlated multi-omic signatures associated with an outcome (32). Regularized regression, random forests, gradient boosting, graph-based learning, and multimodal neural networks may be appropriate depending on sample size, dimensionality, and missingness. Fusion can occur early by concatenating features, late by combining modality-specific predictions, or through intermediate attention-based representations. More complex architectures should only be used when their added performance is demonstrated against transparent baselines.

Multimodal models can combine pathology, radiology, molecular profiles, and functional drug-response phenotypes. Joint histology-genomic approaches have demonstrated that integrated representations can improve outcome prediction while identifying morphology linked to molecular states (33). In the proposed patient-model-algorithm-patient loop, a clinically useful output would not be a single opaque risk score. It would be a calibrated estimate with confidence intervals, the contribution of each modality, visual or feature-level explanations, and a record of how the prediction changes when a modality is absent. Model development should use patient-level or animal-level partitioning, nested cross-validation for tuning, locked external validation, calibration analysis, decision-curve analysis, and sensitivity analyses for batch effects and missing data. Reporting should follow the TRIPOD+AI guidance for clinical prediction models (34).

The most important validation is translational rather than merely statistical. A signature discovered in human tumors should be tested in independent human data and then challenged across organoids, PDXs, GEMMs, canine tumors, or porcine studies according to the mechanism it claims to represent. Conversely, a signature generated in a comparative model should be evaluated for species-specific features before being projected onto human cohorts. An algorithm is useful only when it makes explicit which biological evidence is transferable, which is model-dependent, and what experiment or clinical observation could falsify the prediction.

### Fit-for-purpose model selection and reverse translation

A reverse-translational program begins with an unresolved clinical observation and returns to the clinic only after the mechanism and predictive value have survived progressively different constraints. Human TNBC specimens and spontaneous canine mammary tumors provide clinically annotated biological entry points. Organoids support scalable perturbation, PDXs preserve patient-linked *in vivo* evolution, GEMMs test causality in immunocompetent hosts, and large animals provide human-scale procedural validation. Reverse translation is therefore iterative rather than linear: observations move from patient to model, from model to algorithm, and from integrated evidence back to prospective clinical testing (35).

Model complementarity does not mean that every project requires every platform. The appropriate sequence is determined by the claim. A cell-intrinsic synthetic lethal interaction may progress from organoids to PDXs; an immune-spatial biomarker may require canine tumors and GEMMs before clinical validation; and an image-guided locoregional strategy may justify a porcine study only after biological efficacy is established elsewhere. **Table 1** summarizes this fit-for-purpose logic. The field will advance when model limitations are treated as design variables, computational methods are specified and validated, and claims are restricted to the evidence that each platform can genuinely provide.

**Table 1:** Fit-for-purpose comparative and translational biomodels for TNBC precision medicine.

Model/platform	Biological value	Principal limitations and bias	Best-fit applications	Required validation and AI-compatible outputs
Spontaneous canine mammary tumors	Naturally occurring disease in outbred, immunocompetent hosts; real-world heterogeneity and host-tumor-microenvironment interactions.	Species and breed effects; variable reproductive history, staging, receptor testing, treatment, and follow-up; not all canine triple-negative tumors represent human TNBC.	Comparative biomarker discovery; immune and stromal ecology; longitudinal response studies; veterinary clinical trials.	Harmonized pathology and receptor thresholds; genomic/transcriptomic subtype assignment; linked clinical metadata; pathology, imaging, blood, and outcome data.
Human and canine tumor-derived organoids	Rapid, scalable functional testing with retention of important tumor-cell phenotypes and molecular alterations; compatible with genetic perturbation.	Variable establishment; media and matrix selection; clonal drift; incomplete immune, stromal, vascular, endocrine, and pharmacokinetic context.	Drug-response profiling; combination prioritization; CRISPR-Cas9 screens; resistance evolution; matched-species comparison.	Early-passage concordance with source tumor; standardized assay conditions; replicate dose-response data; microscopy, viability, and multi-omics features.
Patient-derived xenografts	Patient-linked architecture, heterogeneity, metastasis, residual disease, and treatment-selected evolution *in vivo*.	Engraftment bias; long latency and high cost; immunodeficient host; progressive replacement of human stroma by murine stroma.	*In vivo* efficacy; treatment sequence; residual disease and metastatic progression; validation of organoid hits.	Tumor-source and passage tracking; pharmacokinetics; matched pathology and sequencing; longitudinal imaging and response labels.
Genetically engineered mouse models	Controlled lineage, timing, and oncogenic events in an immunocompetent host; strong mechanistic resolution.	Restricted genetic background; engineered-driver dominance; species-specific evolution; variable correspondence to human TNBC subtypes.	Causal testing of drivers; cell of origin; immune mechanisms; initiation, progression, and metastasis.	Cross-species subtype mapping; independent models for the same mechanism; spatial immune profiling and longitudinal response data.
Large-animal and Oncopig platforms	Human-scale anatomy and procedures; serial imaging and biopsy; device, catheter, surgery, and locoregional delivery studies.	The KRAS/TP53 Oncopig is not a validated TNBC model; high cost, low throughput, specialized facilities, limited reagents, and fewer established mammary applications.	Human-scale stress testing of imaging biomarkers, sampling schedules, interventions, and locoregional delivery after disease-specific prioritization.	Mammary-relevant implementation; prespecified procedural endpoints; harmonized imaging; radiological-pathological correlation; serial pharmacokinetic and molecular data.
Multimodal computational layer	Connects pathology, imaging, molecular profiles, serial sampling, and functional drug response across patients and models.	Data leakage, batch effects, domain shift, missing modalities, overfitting, poor calibration, and limited interpretability.	Biomarker prioritization; response prediction; cross-model concordance; hypothesis generation for prospective testing.	Patient-level partitioning; transparent baselines; nested cross-validation; locked external validation; calibration, interpretability, and leave-one-species-out analysis.

## References

[1] Lehmann BD Bauer JA Chen X Sanders ME Chakravarthy AB Shyr Y . Identification of human triple-negative breast cancer subtypes and preclinical models for selection of targeted therapies. J Clin Invest. (2011) 121:2750–67. doi: 10.1172/JCI45014 21633166 PMC3127435

[2] Burstein MD Tsimelzon A Poage GM Covington KR Contreras A Fuqua SAW . Comprehensive genomic analysis identifies novel subtypes and targets of triple-negative breast cancer. Clin Cancer Res. (2015) 21:1688–98. doi: 10.1158/1078-0432.CCR-14-0432 25208879 PMC4362882

[3] Jiang YZ Ma D Suo C Shi J Xue M Hu X . Genomic and transcriptomic landscape of triple-negative breast cancers: subtypes and treatment strategies. Cancer Cell. (2019) 35:428–440.e5. doi: 10.1016/j.ccell.2019.02.001 30853353

[4] Yan Y Lin Y Kumar T Bai S Thennavan A Li J . Ecotypes of triple-negative breast cancer in response to chemotherapy. Nature. (2026) 654:1088–97. doi: 10.1038/s41586-026-10469-9 42129561 PMC13293894

[5] Wang XQ Danenberg E Huang CS Egle D Callari M Bermejo B . Spatial predictors of immunotherapy response in triple-negative breast cancer. Nature. (2023) 621:868–76. doi: 10.1038/s41586-023-06498-3 37674077 PMC10533410

[6] Dent R Trudeau M Pritchard KI Hanna WM Kahn HK Sawka CA . Triple-negative breast cancer: clinical features and patterns of recurrence. Clin Cancer Res. (2007) 13:4429–34. doi: 10.1158/1078-0432.CCR-06-3045 17671126

[7] Liedtke C Mazouni C Hess KR Andre F Tordai A Mejia JA . Response to neoadjuvant therapy and long-term survival in patients with triple-negative breast cancer. J Clin Oncol. (2008) 26:1275–81. doi: 10.1200/JCO.2007.14.4147 18250347

[8] Schmid P Cortes J Dent R McArthur H Pusztai L Kummel S . Overall survival with pembrolizumab in early-stage triple-negative breast cancer. N Engl J Med. (2024) 391:1981–91. doi: 10.1056/NEJMoa2409932 39282906

[9] Cortes J Rugo HS Cescon DW Im SA Yusof MM Gallardo C . Pembrolizumab plus chemotherapy in advanced triple-negative breast cancer. N Engl J Med. (2022) 387:217–26. doi: 10.1056/NEJMoa2202809 35857659

[10] Tutt ANJ Garber JE Kaufman B Viale G Fumagalli D Rastogi P . Adjuvant olaparib for patients with BRCA1- or BRCA2-mutated breast cancer. N Engl J Med. (2021) 384:2394–405. doi: 10.1056/NEJMoa2105215 34081848 PMC9126186

[11] Masuda N Lee SJ Ohtani S Im YH Lee ES Yokota I . Adjuvant capecitabine for breast cancer after preoperative chemotherapy. N Engl J Med. (2017) 376:2147–59. doi: 10.1056/NEJMoa1612645 28564564

[12] Bardia A Hurvitz SA Tolaney SM Loirat D Punie K Oliveira M . Sacituzumab govitecan in metastatic triple-negative breast cancer. N Engl J Med. (2021) 384:1529–41. doi: 10.1056/NEJMoa2028485 33882206

[13] Liu D Xiong H Ellis AE Northrup NC Rodriguez CO Jr O'Regan RM . Molecular homology and difference between spontaneous canine mammary cancer and human breast cancer. Cancer Res. (2014) 74:5045–56. doi: 10.1158/0008-5472.CAN-14-0392 25082814 PMC4167563

[14] Kim TM Yang IS Seung BJ Lee S Kim D Ha YJ . Cross-species oncogenic signatures of breast cancer in canine mammary tumors. Nat Commun. (2020) 11:3616. doi: 10.1038/s41467-020-17458-0 32680987 PMC7367841

[15] Watson J Wang T Ho KL Feng Y Mahawan T Dobbin KK . Human basal-like breast cancer is represented by one of the two mammary tumor subtypes in dogs. Breast Cancer Res. (2023) 25:114. doi: 10.1186/s13058-023-01705-5 37789381 PMC10546663

[16] Sachs N de Ligt J Kopper O Gogola E Bounova G Weeber F . A living biobank of breast cancer organoids captures disease heterogeneity. Cell. (2018) 172:373–386.e10. doi: 10.1016/j.cell.2017.11.010 29224780

[17] Inglebert M Dettwiler M Hahn K Letko A Drogemuller C Doench J . A living biobank of canine mammary tumor organoids as a comparative model for human breast cancer. Sci Rep. (2022) 12:18051. doi: 10.1038/s41598-022-21706-2 36302863 PMC9614008

[18] Guillen KP Fujita M Butterfield AJ Scherer SD Bailey MH Chu Z . A human breast cancer-derived xenograft and organoid platform for drug discovery and precision oncology. Nat Cancer. (2022) 3:232–50. doi: 10.1038/s43018-022-00337-6 35221336 PMC8882468

[19] DeRose YS Wang G Lin YC Bernard PS Buys SS Ebbert MTW . Tumor grafts derived from women with breast cancer authentically reflect tumor pathology, growth, metastasis and disease outcomes. Nat Med. (2011) 17:1514–20. doi: 10.1038/nm.2454 22019887 PMC3553601

[20] Bruna A Rueda OM Greenwood W Batra AS Callari M Batra RN . A biobank of breast cancer explants with preserved intra-tumor heterogeneity to screen anticancer compounds. Cell. (2016) 167:260–274.e22. doi: 10.1016/j.cell.2016.08.041 27641504 PMC5037319

[21] Herschkowitz JI Simin K Weigman VJ Mikaelian I Usary J Hu Z . Identification of conserved gene expression features between murine mammary carcinoma models and human breast tumors. Genome Biol. (2007) 8:R76. doi: 10.1186/gb-2007-8-5-r76 17493263 PMC1929138

[22] Pfefferle AD Herschkowitz JI Usary J Harrell JC Spike BT Adams JR . Transcriptomic classification of genetically engineered mouse models of breast cancer identifies human subtype counterparts. Genome Biol. (2013) 14:R125. doi: 10.1186/gb-2013-14-11-r125 24220145 PMC4053990

[23] Schook LB Collares TV Hu W Liang Y Rodrigues FM Rund LA . A genetic porcine model of cancer. PloS One. (2015) 10:e0128864. doi: 10.1371/journal.pone.0128864 26132737 PMC4488487

[24] Donninger H Hobbing K Schmidt ML Walters E Rund L Schook L . A porcine model system of BRCA1 driven breast cancer. Front Genet. (2015) 6:269. doi: 10.3389/fgene.2015.00269 26379698 PMC4548227

[25] Mondal P Bailey KL Cartwright SB Band V Carlson MA . Large animal models of breast cancer. Front Oncol. (2022) 12:788038. doi: 10.3389/fonc.2022.788038 35186735 PMC8855936

[26] Boas FE Nurili F Bendet A Cheleuitte-Nieves C Basturk O Askan G . Induction and characterization of pancreatic cancer in a transgenic pig model. PloS One. (2020) 15:e0239391. doi: 10.1371/journal.pone.0239391 32956389 PMC7505440

[27] Lu MY Williamson DFK Chen TY Chen RJ Barbieri M Mahmood F . Data-efficient and weakly supervised computational pathology on whole-slide images. Nat BioMed Eng. (2021) 5:555–70. doi: 10.1038/s41551-020-00682-w 33649564 PMC8711640

[28] Shamai G Livne A Polonia A Sabo E Cretu A Bar-Sela G . Deep learning-based image analysis predicts PD-L1 status from H&E-stained histopathology images in breast cancer. Nat Commun. (2022) 13:6753. doi: 10.1038/s41467-022-34275-9 36347854 PMC9643479

[29] Rahaman MM Millar EKA Meijering E . Breast cancer histopathology image-based gene expression prediction using spatial transcriptomics data and deep learning. Sci Rep. (2023) 13:13604. doi: 10.1038/s41598-023-40219-0 37604916 PMC10442349

[30] Xie T Gong J Zhao Q Wu C Wu S Peng W . Development and validation of peritumoral vascular and intratumoral radiomics to predict pathologic complete responses to neoadjuvant chemotherapy in patients with triple-negative breast cancer. BMC Med Imaging. (2024) 24:136. doi: 10.1186/s12880-024-01311-7 38844842 PMC11155097

[31] Argelaguet R Arnol D Bredikhin D Deloro Y Velten B Marioni JC . MOFA+: a statistical framework for comprehensive integration of multi-modal single-cell data. Genome Biol. (2020) 21:111. doi: 10.1186/s13059-020-02015-1 32393329 PMC7212577

[32] Singh A Shannon CP Gautier B Rohart F Vacher M Tebbutt SJ . DIABLO: an integrative approach for identifying key molecular drivers from multi-omics assays. Bioinformatics. (2019) 35:3055–62. doi: 10.1093/bioinformatics/bty1054 30657866 PMC6735831

[33] Mobadersany P Yousefi S Amgad M Gutman DA Barnholtz-Sloan JS Vega JEV . Predicting cancer outcomes from histology and genomics using convolutional networks. Proc Natl Acad Sci USA. (2018) 115:E2970–9. doi: 10.1073/pnas.1717139115 29531073 PMC5879673

[34] Collins GS Moons KGM Dhiman P Riley RD Beam AL Van Calster B . TRIPOD+AI statement: updated guidance for reporting clinical prediction models that use regression or machine learning methods. BMJ. (2024) 385:e078378. doi: 10.1136/bmj-2023-078378 38626948 PMC11019967

[35] Shakhnovich V . It's time to reverse our thinking: the reverse translation research paradigm. Clin Transl Sci. (2018) 11:98–9. doi: 10.1111/cts.12538 29423973 PMC5866972

